# Multifocal electroretinography assessment of retinal responses to myopic defocus lenses

**DOI:** 10.7717/peerj.21766

**Published:** 2026-10-05

**Authors:** Mohammed Alhazmi, Mohammed Althomali, Muteb Alanazi, Wafa Alotaibi, Eman Alghamdi, Ali Almustanyir, Fahd Alqahtani, Maria Liu

**Affiliations:** 1 Optometry Department, College of Applied Medical Sciences, King Saud University, Riyadh, Saudi Arabia; 2 Optometry Department, East Jeddah General Hospital, Jeddah, Makkah, Saudi Arabia; 3 Ophthalmology Department, King Saud University Medical City, Riyadh, Saudi Arabia; 4 School of Optometry, University of California, Berkeley, CA, United States of America

**Keywords:** Multifocal electroretinogram, Myopia control, Myopia control lenses, Regional retinal variability, Retinal responses

## Abstract

**Aim:**

To evaluate the short-term retinal electrical responses elicited by defocus segment lenses in emmetropic eyes using multifocal electroretinography (mfERG).

**Materials & Methods:**

A prospective cross-sectional study enrolled 14 young, healthy emmetropic participants aged 18 to 22 years with no ocular or systemic diseases. Comprehensive eye examinations were conducted, and mfERG recordings were obtained using six different condition scenarios. Retinal responses were analyzed across hexagon ring groupings defined by angular extent (Ring 1: 2.5°, Ring 2: 7.5°, Ring 3: 14°, Ring 4: 22°, Ring 5: 30°). Statistical analysis was performed to compare retinal responses across different lens types and retinal regions.

**Results:**

No significant differences in retinal electrical responses were detected across the six lens conditions for negative (N1) latency (ms), positive (P1) latency (ms), or P1 amplitude (nV/deg^2^) (all *p* > 0.05). Regional variations in amplitude and latency across concentric retinal rings were observed but reflected expected normative mfERG gradients independent of lens type.

**Conclusion:**

The tested defocus segment lens designs did not produce statistically detectable alterations in first order mfERG parameters in structurally intact emmetropic eyes under brief exposure. Although the analytical framework demonstrated sufficient sensitivity to identify medium to large electrophysiological effects, the current design may lack resolution for subtle retinal adaptations. Future investigations incorporating larger cohorts, prolonged optical exposure, and nonlinear analytical techniques in ametropic populations will be necessary to determine whether clinically relevant myopia control mechanisms correspond to measurable electrophysiological modulation.

## Introduction

Myopia has emerged as a significant global public health concern, with projections indicating that its prevalence will reach approximately five billion cases worldwide by 2050, including 1 billion cases of high myopia (Holden et al., 2016). This alarming rise not only underscores the growing burden of refractive errors but also highlights the increased risk of severe ocular pathologies associated with myopia, such as retinal detachment (Bullimore et al., 2021; Ludwig et al., 2023), glaucoma (Ha et al., 2022), other vision-threatening conditions. These complications pose substantial challenges to individuals’ quality of life and healthcare systems globally.

To mitigate the potential societal and economic burdens posed by myopia, extensive research efforts have been directed toward identifying its underlying causes (Baird et al., 2020; Huang, Chang & Wu, 2015; Russo et al., 2022; Yu et al., 2023), and developing interventions to slow its progression (Baird et al., 2020; Bao et al., 2022; Bullimore et al., 2021; Hiraoka, 2022; Lam et al., 2020; Remon et al., 2020; Russo et al., 2022; Trier et al., 2023; Zhao et al., 2020).

Among these interventions, optical strategies have gained considerable attention due to their non-invasive nature and promising clinical outcomes. Notably, the advent of defocus segment lenses, such as defocus incorporated multiple segments (DIMS) (Lam et al., 2020) and highly aspherical lenslet target (H.A.L.T.) designs (Bao et al., 2022) marks a significant advancement in myopia management strategies. Clinical studies have demonstrated that DIMS lenses can reduce myopia progression by 52% and axial elongation by 62% (Lam et al., 2020), while lenslets with differential asphericity have been shown to decrease myopia progression by 67% and axial elongation by 64% (Bao et al., 2022). These findings underscore the efficacy of defocus segment lenses as a viable strategy for slowing myopia progression, particularly in pediatric populations.

The hypothesis that defocus segment lenses might elicit measurable short-term retinal electrophysiological changes is grounded in evidence demonstrating the rapid change in retinal response to optical defocus as reported in previous studies (Chan & Siu, 2003). Previous research has shown that even brief exposures to myopic or hyperopic defocus can induce transient alterations at both the retinal (Chin et al., 2015; Ho et al., 2012), and choroidal levels (Delshad et al., 2020; Huang et al., 2025). Multifocal electroretinography (mfERG) studies have reported rapid modulations in retinal responses under defocus conditions, suggesting an immediate neural adaptation to blurred imagery (Chin et al., 2015; Ho et al., 2012). Concurrently, optical coherence tomography investigations have documented short-term shifts in choroidal thickness in response to defocus, indicating a complex, interactive signaling cascade between the retina and the choroid (Delshad et al., 2020; Huang et al., 2025). Despite these encouraging physiological findings, critical gaps remain regarding how modern defocus segment lenses specifically influence retinal electrophysiology, particularly whether their spatially targeted optical profiles elicit regionally distinct responses.

The primary rationale for employing mfERG in this investigation lies in the optical architecture of the lenses under study. Defocus segment lenses impose non-uniform, spatially distributed optical signals across the retina while preserving central foveal clarity. Consequently, any electrophysiological adaptation induced by these lenses is expected to be topographically specific rather than global. Full-field electroretinography (ERG) integrates responses across the entire stimulated retina, which would inherently obscure localized, eccentricity-dependent modulation. In contrast, mfERG provides the spatial resolution necessary to isolate and quantify retinal function across distinct concentric zones, enabling precise topographical mapping of neural responses to spatially targeted defocus profiles (Azarmina, 2013; Hoffmann et al., 2021; Hood et al., 2003; Hood et al., 1997). Two commercially available defocus segment spectacle lenses were investigated: the MiyoSmart (Hoya) lens and the Stellest (Essilor) lens. The MiyoSmart lens incorporates defocus incorporated multiple segments (DIMS), consisting of a central nine mm diameter zone for clear distance vision surrounded by approximately 400 +3.00D defocus segments arranged in a hexagonal pattern (Lam et al., 2020). The Stellest lens uses highly aspherical lenslet target (H.A.L.T.) technology, featuring a constellation of +3.50D aspherical lenslets (Bao et al., 2022). Given that these lenslet arrays project defocus signals to specific peripheral eccentricities (approximately 15°–30° of visual angle), regional variations in retinal responses are anticipated, particularly in areas corresponding to the optical treatment zones.

Based on established electrophysiological literature demonstrating that acute myopic defocus transiently suppresses outer retinal drive and delays neural processing, a directional primary hypothesis was formulated. Defocus segment lenses are expected to elicit a measurable reduction in P1 amplitude and a prolongation of P1 latency in the peripheral retinal rings corresponding to the projected defocus zones relative to the central clear zones and the no lens control condition. This expectation aligns with prior mfERG investigations reporting sign dependent modulation of retinal responses under blurred optical conditions, wherein reduced image contrast and spatial frequency degradation attenuate photoreceptor to bipolar cell signaling efficiency and increase response synchronization time (Chin et al., 2015; Ho et al., 2012). The secondary hypothesis posits that the magnitude of these peripheral modulations will vary according to the distinct spatial distribution of defocus signals inherent to each optical design. To test these hypotheses, mfERG responses were evaluated across six experimental conditions and stratified into five concentric retinal rings.

Accordingly, the aim of this study is to evaluate the short-term retinal electrical responses elicited by defocus segment lenses in emmetropic eyes using mfERG.

## Materials & Methods

### Study design and subjects

This cross-sectional study enrolled 14 visually healthy young adults (six males, eight females) aged 18 to 22 years (mean ± SD: 20 ± 1.46). Ethical approval was granted by the Institutional Review Board at King Saud University in accordance with the Declaration of Helsinki (approval number E-23-7783). Written informed consent was obtained from all participants before study initiation, and confidentiality was ensured to maintain participant anonymity.

Participants were included if they exhibited a spherical equivalent (SE) within the range of −0.50D to +0.50D and an uncorrected visual acuity (VA) of 6/6 or better. Exclusion criteria encompassed the presence of ocular or systemic disorders, a history of ocular or systemic trauma, current or past use of ocular or systemic medications, and impaired binocular vision. Additionally, participants with refractive parameters outside the specified SE range or with an uncorrected VA worse than 6/6 were excluded.

### Ocular measurements

All participants underwent a series of standard ophthalmic evaluations, including subjective refraction, slit-lamp biomicroscopy (Haag-Streit BQ 900), and axial length assessment *via* noncontact partial coherence interferometry (IOLMaster^®^ 500, ZEISS). Accommodation measurements were conducted using the push-up method and an open-field autorefractometer (Grand Seiko WAM-5500).

### Data collection

The mfERG recordings were obtained using the Diagnosys mfERG system (Diagnosys LLC, Lowell, MA) following the manufacturer’s protocol. The mfERG stimulus array consisted of 61 non-scaled hexagons subtending 60 degrees horizontally and vertically, presented at a working distance of 33 cm (Fig. 1). The stimulus was displayed on a 32-inch LCD monitor with a brightness of 1,000 cd/m^2^ and a contrast ratio of 1,000:1. Each hexagon alternated between bright and dark states, following a pseudo-random binary m-sequence stimulation protocol.

**Figure 1:**
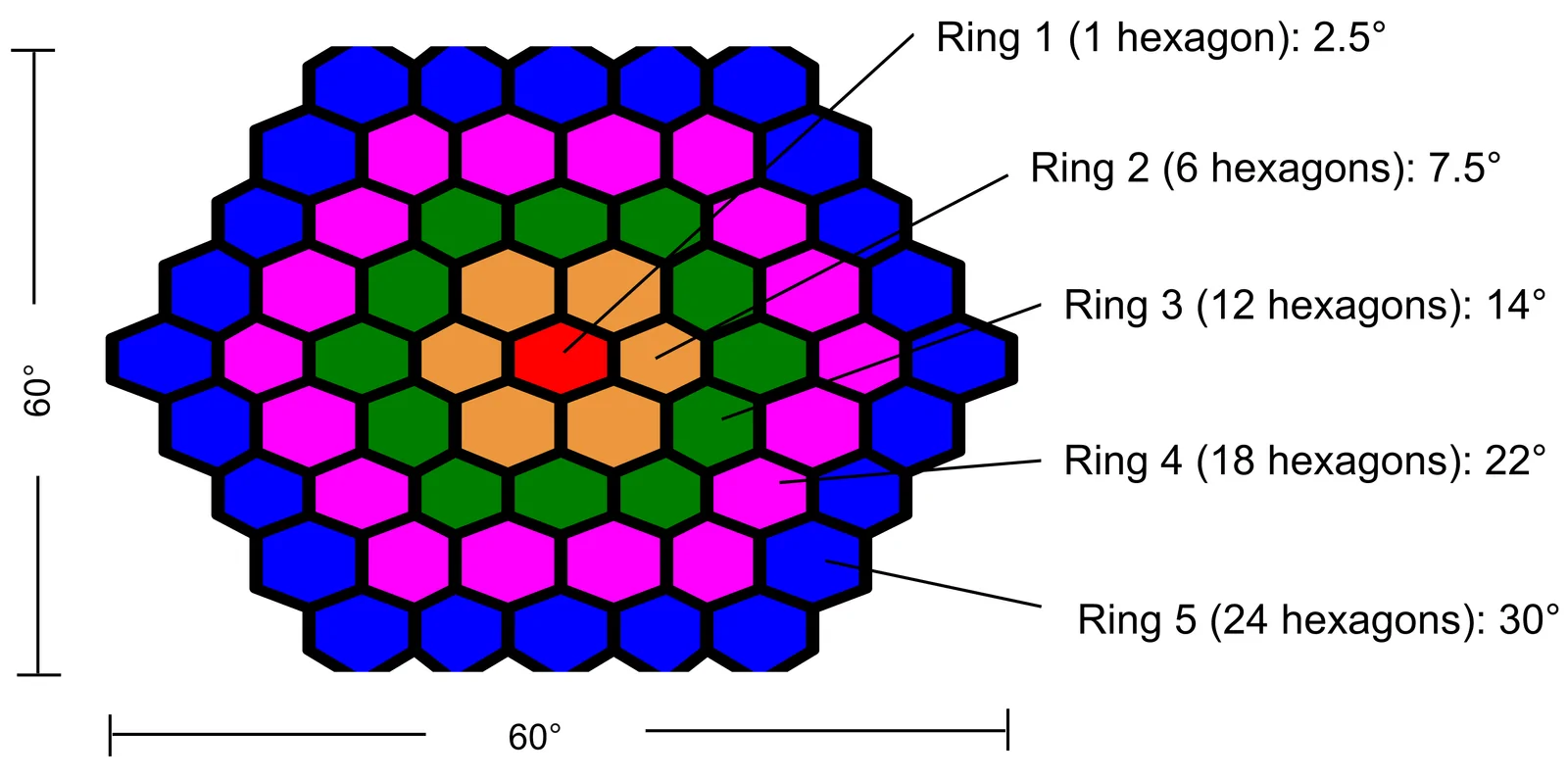
The mfERG stimulus array consisted of 61 hexagons arranged in five concentric rings, each defined by angular distance from fixation. Ring 1 contained 1 hexagon subtending 2.5°, Ring 2 contained 6 hexagons subtending 7.5°, Ring 3 contained 12 hexagons subtending 14°, Ring 4 contained 18 hexagons subtending 22°, and Ring 5 contained 24 hexagons subtending 30° . The total stimulus field extended 30° in all directions (60° horizontally and vertically).

Figure 2 shows the defocus profiles of the tested lens designs and their projected retinal locations, mapped according to the hexagonal mfERG stimulus array.

**Figure 2:**
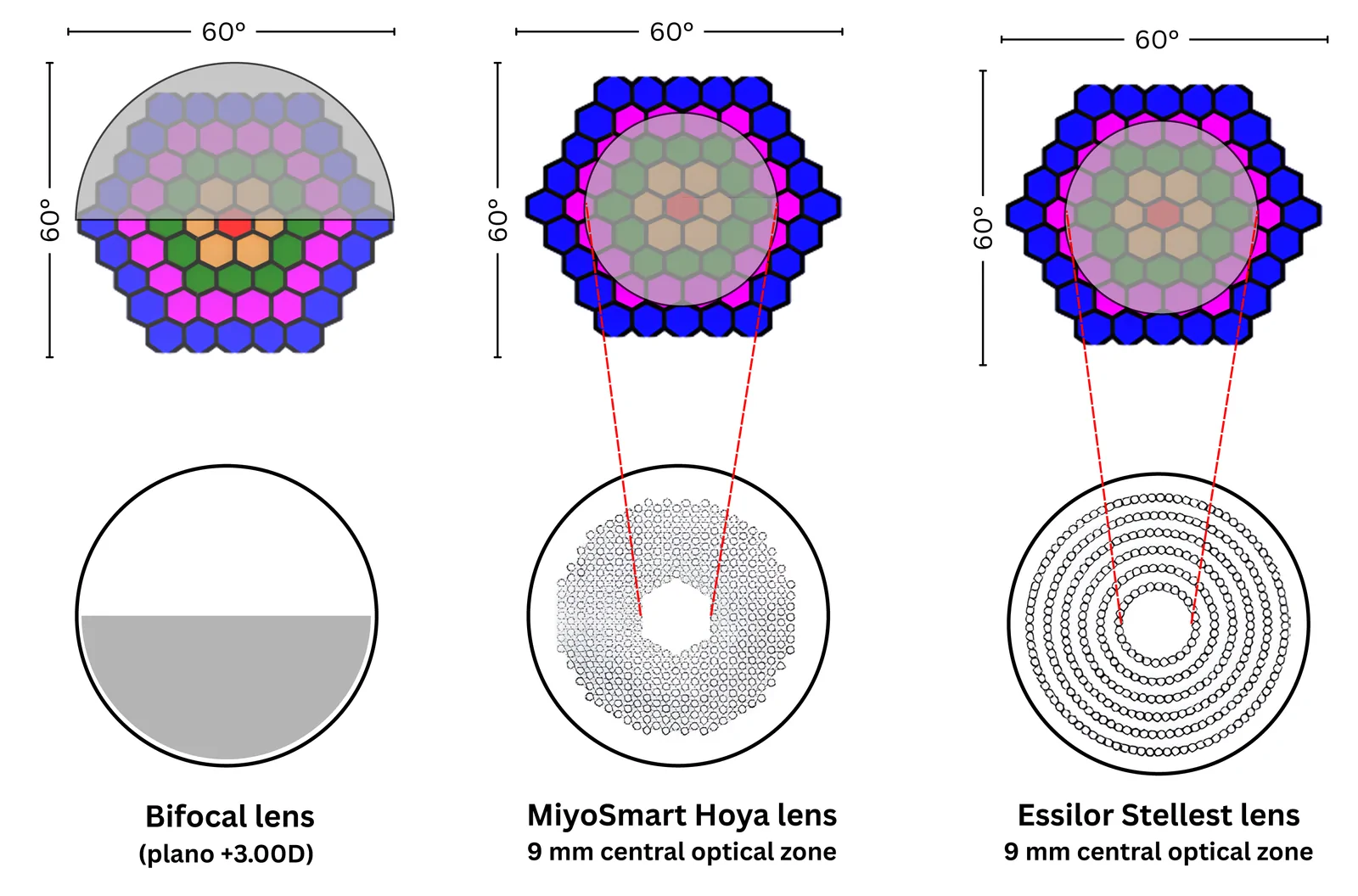
Schematic illustration of the optical designs of the three myopia control spectacle lens designs investigated in this study, with shaded areas indicating the field of full illumination projected onto the retina. The shaded areas represent the field of full illumination for the bifocal defocus zone and the nine mm central zone projected onto the retina by MiyoSmart Hoya lens and the Stellest Essilor lens.

Recordings were obtained once for each participant, under each of the six different experimental conditions: (1) no lens, (2) bifocal lens (plano +3.00D), (3) MiyoSmart Hoya lens (0.00D), (4) Essilor Stellest lens (0.00D), (5) MiyoSmart Hoya lens (−3.00D) and (6) Essilor Stellest lens (−3.00D). The sequence of conditions was randomized to minimize order effects. Each mfERG recording session lasted less than 7 min, and a 10-minute break was provided after the third session.

Three adhesive electrodes were positioned on the participants’ heads (center of the forehead and at the medial and lateral canthi). DTL electrodes (NEUROSPEC AG, Stan, Switzerland) were placed on the lower corneal margin of each eye following the instillation of proparacaine hydrochloride ophthalmic solution (0.5%) for corneal anesthesia.

Pupillary dilation was induced using topical 2.5% phenylephrine hydrochloride ophthalmic solution to facilitate ocular imaging. Two drops were administered to each eye at 5-minute intervals. This agent was selected for its mydriatic properties while preserving ciliary muscle function (Richdale et al., 2012), thereby allowing natural accommodative activity to remain intact throughout the experimental session. Maintaining active accommodation was a deliberate design choice to simulate physiological viewing conditions. Amplitude of accommodation and accommodative response were performed at two predetermined time points: immediately prior to the initial phenylephrine instillation and 30 min following the drop administration.

### Statistical analysis

Statistical analysis was performed using SPSS software version 29.0.1 (IBM SPSS Statistics Inc., Chicago, IL, USA). Descriptive statistics, including means and standard deviations (SD), were computed for all quantitative variables. Retinal response data were stratified into five concentric hexagon rings based on angular distance from fixation: Ring 1 (2.5°), Ring 2 (7.5°), Ring 3 (14°), Ring 4 (22°), and Ring 5 (30°) (Fig. 1). To preserve statistical independence and avoid inter-eye correlation, only one eye per participant was included in the analyses, with the right eye designated as the study eye. Preliminary inter-eye comparisons were conducted across all experimental conditions to confirm the absence of systematic differences. To directly test the primary hypothesis regarding lens specific effects on retinal electrophysiology, a two way repeated measures analysis of variance (ANOVA) was employed for each outcome variable: N1 latency (ms), P1 latency (ms), and P1 amplitude (nV/deg^2^). This model incorporated two within subject factors: lens condition (six levels: no lens, bifocal, MiyoSmart 0.00D, MiyoSmart 3.00D, Stellest 0.00D, and Stellest 3.00D) and retinal ring (five levels: Rings 1 to 5). The main effect of lens condition directly addressed whether defocus segment lenses differentially modulated retinal responses compared to the control condition. The main effect of retinal ring evaluated normative topographical gradients, while the lens condition by retinal ring interaction assessed whether lens specific optical designs produced eccentricity dependent modulation of electrophysiological parameters. Assumptions of normality were verified using the Shapiro-Wilk test, and sphericity was assessed *via* the Mauchly test. Where the assumption of sphericity was violated, Greenhouse Geisser correction was applied to degrees of freedom and adjusted *p* values were reported. In the event of statistically significant main effects or interactions, *post hoc* pairwise comparisons were conducted using Tukey honestly significant difference (HSD) correction to control for multiple testing.

The stability of accommodative function following phenylephrine instillation was assessed by comparing pre- and post-administration accommodative response and amplitude of accommodation using paired-samples *t*-tests (two-tailed, *α* = 0.05).

Statistical significance was considered for *p* values <0.05 in all analyses.

## Results

### Demographic characteristics

Demographic and ocular characteristics, including age, spherical equivalent, axial length, and accommodation parameters, are reported in Table 1.

**Table 1:** Baseline demographic and ocular parameters (*N* = 14); values expressed as mean ± SD and range.

**Variable**	**(Mean ± SD)**	***Range***
Age (years)	20 ± 1.52	18–22
Spherical Equivalent (D)	0.09 ± 0.37	−0.50 - +0.50
Axial Length (mm)	23.48 ± 0.78	25.41–22.42
Accommodative response (D)	+2.25 ± 0.39	+1.50 - +2.75
Amplitude of accommodation (D)	+12.40 ± 1.37	+10.38 - +14.13

No statistically significant differences were detected for right eye accommodative response (t(13) = 0.78, *p* = 0.45) or amplitude of accommodation (t(13) = 0.92, *p* = 0.37), confirming that the 2.5% phenylephrine protocol preserved accommodative function during testing.

### Retinal electrophysiological responses to defocus segment lenses

A two-way repeated measures ANOVA was employed to evaluate the effects of lens condition and retinal ring on mfERG outcome measures. Across all outcome measures, the main effect of lens condition was not statistically significant for N1 latency (*p* = 0.260, *ηp*^2^ = 0.097), P1 latency (*p* = 0.288, *ηp*^2^ = 0.090), or P1 amplitude (Greenhouse Geisser corrected *p* = 0.061, *ηp*^2^ = 0.160), indicating that the tested defocus segment lens designs did not produce measurable alterations in retinal electrophysiological function under the present experimental conditions (Fig. 3). Furthermore, the lens condition by retinal ring interaction was not statistically significant for any outcome (N1 latency: *p* = 0.229, *ηp*^2^ = 0.097; P1 latency: *p* = 0.590, *ηp*^2^ = 0.057; P1 amplitude: *p* = 0.111, *ηp*^2^ = 0.132), demonstrating that the spatial distribution of retinal responses across central and peripheral regions was not differentially modulated by lens condition.

**Figure 3:**
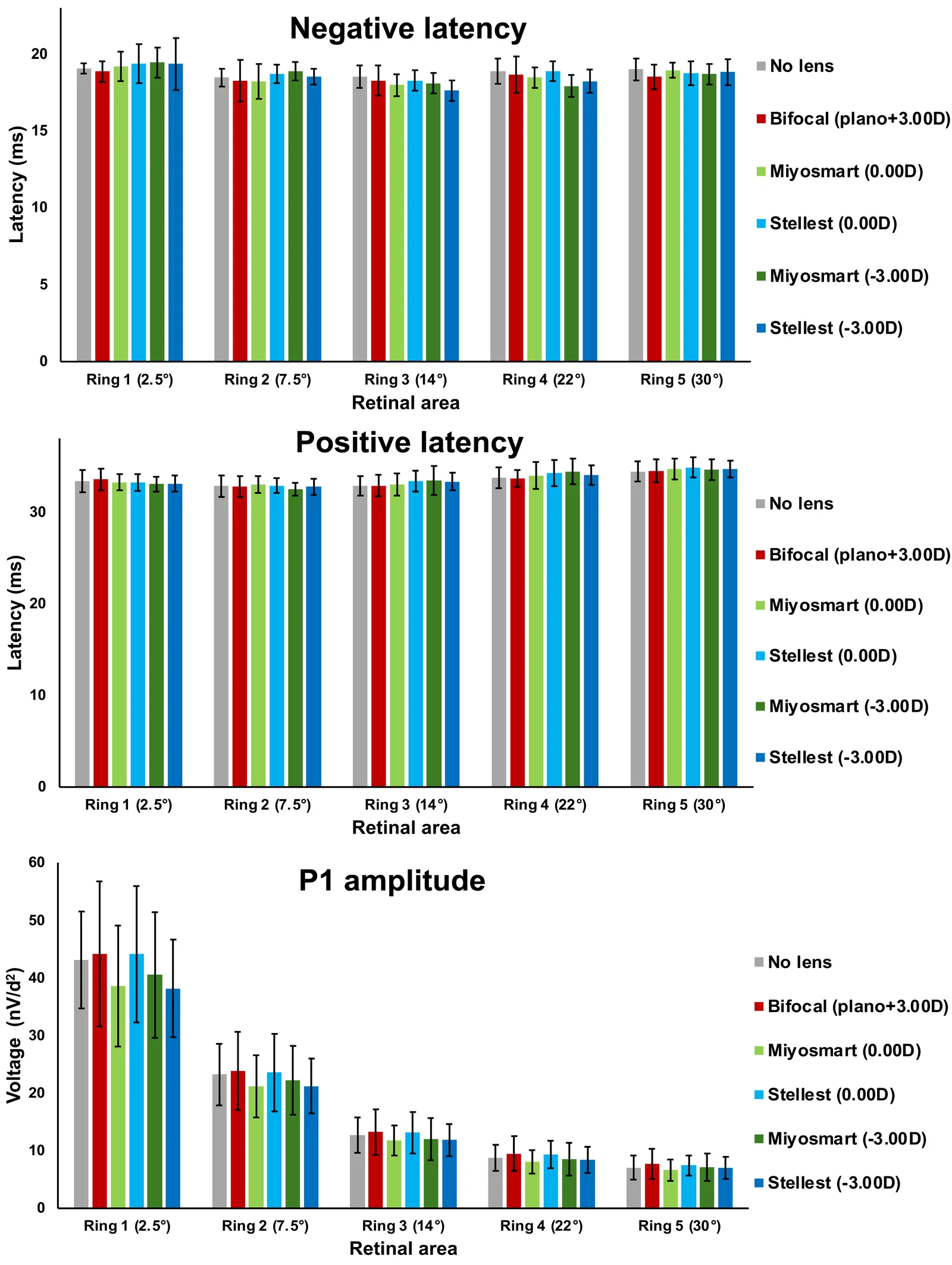
Mean retinal electrical activities (N1 latency, P1 latency, and P1 amplitude) across six experimental lens conditions and five concentric retinal rings. The tested defocus segment lens designs did not differentially modulate retinal electrophysiological function, as indicated by the absence of significant differences between lens conditions and a not significant lens condition by retinal ring interaction. Expected physiological gradients across retinal eccentricities were present but remained unchanged across all optical treatments. Error bars represent SD.

In confirmatory analysis, a robust main effect of retinal ring was observed across all outcome measures (all *p* < 0.001), with large effect sizes (*ηp*^2^ = 0.655 to 0.949), consistent with well established physiological gradients in mfERG responses across retinal eccentricity. These expected regional variations were present regardless of lens condition, underscoring that the primary finding of this investigation is the absence of additional lens specific modulation beyond normative retinal properties (Fig. 3). *Post hoc* comparisons (Tukey HSD) for statistically significant differences only are provided in Table S1.

## Discussion

The escalating global prevalence of myopia, projected to affect nearly five billion individuals by 2050 (Holden et al., 2016), has intensified efforts to develop effective interventions. Defocus segment lenses, such as DIMS (Lam et al., 2020) and aspherical lenslets (Bao et al., 2022), have demonstrated efficacy in slowing axial elongation in myopic eyes. However, their impact on retinal electrophysiology remains understudied. This investigation addresses this gap by evaluating mfERG responses in emmetropic eyes under varied defocus conditions.

Our primary finding is the absence of significant differences in retinal electrophysiological responses across the six lens conditions. While regional variability in amplitude and latency was observed across the posterior pole, these gradients reflect well-established normative mfERG properties (Alanazi, 2023; Wagner & Strasser, 2023) and remained independent of optical treatment. Collectively, these results demonstrate that defocus segment lenses do not acutely alter retinal function in structurally intact emmetropic eyes, establishing a critical baseline for future investigations in myopic populations.

A fundamental methodological choice in this study was the recruitment of emmetropic participants to isolate the lens effects from confounding factors inherent to myopia. These confounders include significant axial length variability, alterations in posterior ocular shape (Matsumura, Kuo & Saw, 2019), and myopia-related structural changes such as retinal thinning and choroidal vascular remodeling (Liu et al., 2022; Wang et al., 2022; Yazdani et al., 2021), all of which are known to disrupt electrophysiological signaling (Gupta, Chakraborty & Verkicharla, 2022; Huang et al., 2023; Kader, 2012). Furthermore, the variable posterior ocular shape and history of optical correction in myopes may lead to neural adaptations that obscure lens-specific effects (Gupta, Chakraborty & Verkicharla, 2022). By contrast, emmetropes maintain a more uniform retinal architecture and lack significant preexisting pathology, ensuring that any observed responses would more purely reflect the lens-induced defocus. This approach of establishing a stable baseline in healthy eyes is a critical precursor to interpreting the more complex responses expected in myopic populations.

The inclusion of the executive bifocal lens serves two primary purposes. First, it represents a clinically validated intervention with demonstrated efficacy in slowing myopia progression in randomized controlled trials (Cheng et al., 2010; Cheng et al., 2014). Second, its optical architecture differs fundamentally from that of contemporary segmented designs: whereas the MiyoSmart and Stellest lenses generate myopic defocus through an array of discrete, spatially discontinuous lenslets, the executive bifocal delivers a continuous, uniform zone of defocus across the entire lower visual field. By incorporating this traditional design alongside modern segmented lenses, we aimed to directly compare retinal electrophysiological responses to continuous *versus* fragmented defocus topographies. This comparative framework enables assessment of whether the retina differentially processes spatially continuous *versus* discontinuous optical signals, thereby providing critical insight into how distinct defocus configurations engage, or fail to engage, retinal adaptation mechanisms.

The regional variations in mfERG responses observed in this study align with normative data describing outer retinal function across the posterior pole (Alanazi, 2023; Wagner & Strasser, 2023). Importantly, these expected physiological gradients were present regardless of lens condition, underscoring that the primary finding of this study is the absence of additional lens-specific modulation beyond these baseline retinal properties. Prior investigations utilizing standard and global flash mfERG paradigms have consistently demonstrated that acute myopic defocus induces sign dependent electrophysiological adaptations, typically manifesting as suppressed P1 amplitudes and prolonged implicit times in retinal regions exposed to blurred imagery (Chin et al., 2015; Ho et al., 2012). Grounded in these findings, the directional framework of the present investigation anticipated that the peripheral defocus segments of the tested spectacle lenses would elicit reduced P1 amplitudes and delayed P1 latencies in peripheral rings compared to central vision and clear field controls. However, the present study revealed no statistically significant directional shifts in any measured parameter across lens conditions. Rather than indicating a failure to detect change, this null result suggests that structural or functional adaptations inherent to myopic eyes such as altered dopamine signaling (Huang et al., 2023) or choroidal changes (Liu et al., 2022; Wang et al., 2022; Yazdani et al., 2021) may be necessary to elicit measurable electrophysiological responses to defocus segment optics.

Defocus segment lenses likely slow axial elongation through complex interactions between optical defocus and retinal-choroidal pathways. However, this study’s findings imply that emmetropic eyes, lacking myopia-associated adaptations, may not exhibit the same sensitivity to lens-induced signals. This aligns with research showing that myopic eyes have heightened responsiveness to peripheral defocus (Gupta, Chakraborty & Verkicharla, 2022), suggesting that lens efficacy depends on preexisting ocular pathology. Future studies combining mfERG with advanced imaging modalities, such as optical coherence tomography (OCT) angiography, could clarify how structural and vascular changes in myopia modulate treatment outcomes.

The absence of significant lens-specific effects likely stems from the physiological stability of the emmetropic retina combined with the analytical focus on linear response (first-order kernel) components. While nonlinear adaptive processing, mediated by inner retinal circuits and assessable *via* second-order kernel analysis or global flash paradigms (Hoffmann et al., 2021; Hood et al., 2008; Hood et al., 2003) may offer enhanced sensitivity to defocus-induced modulation, recent studies have shown that myopia control lenses can modify inner retinal responses, as evidenced by changes in the induced component of the global flash mfERG (Amorim-de Sousa et al., 2024; Turnbull, Goodman & Phillips, 2023). Notably, these inner retinal adaptations were observed in ametropic eyes, which possess structural and functional alterations absent in emmetropes (Gupta, Chakraborty & Verkicharla, 2022; Huang et al., 2023). Consequently, the absence of first-order effects in this emmetropic population suggests that such adaptive mechanisms remain inactive in structurally intact eyes under short-term exposure. While multi-kernel analyses may offer enhanced sensitivity to inner retinal modulations in cohorts with altered signaling pathways, the current findings indicate that standard first-order mfERG adequately captures retinal responsiveness in healthy populations. Future investigations should therefore prioritize multi-kernel analysis in myopic cohorts, where altered retinal signaling pathways are active and expected to demonstrate measurable changes in both linear and nonlinear response components.

Although the study included only 14 participants, the sample size was supported by a priori power analysis (G*Power; effect size *d* = 0.85, power = 0.70) as sufficient to detect physiologically relevant differences in mfERG responses. A repeated-measures design was employed, in which each participant served as their own control across all six lens conditions, reducing inter-subject variability and enhancing statistical sensitivity. To avoid potential bias due to inter-eye correlation, only one eye per participant was included in the analysis. Nevertheless, the current cohort size may limit the capacity to resolve subtle neural adaptations. Accordingly, the absence of statistically significant differences between lens conditions should be interpreted cautiously, as no detectable acute modulation was observed within the analytical constraints of the current protocol, and minor physiologically relevant effects cannot be excluded. These subtle changes may require larger cohorts, extended exposure durations, or higher sensitivity analytical paradigms to resolve. It is also worth noting that recruitment of young adults meeting strict emmetropia criteria presented practical challenges, reinforcing the rationale for the selected cohort size.

The absence of significant changes in accommodative amplitude and response following phenylephrine administration confirms that the mydriatic protocol preserved natural accommodative function throughout the testing session. Unlike cycloplegic agents that paralyze the ciliary muscle, 2.5% phenylephrine acts selectively on the iris dilator muscle *via α*_1_-adrenergic receptors, producing pupillary dilation without clinically meaningful effects on accommodation (Richdale et al., 2012). By evaluating emmetropic participants with uncorrected 6/6 vision, this study eliminated refractive correction-related confounds and captured optical performance under physiologically active conditions. Maintaining intact accommodation enhances ecological validity, ensuring that the measured visual and optical metrics reflect real-world viewing dynamics rather than pharmacologically constrained states. This methodological approach balances experimental control with physiological realism, supporting the translational relevance of the findings.

However, the exclusion of myopes restricts the generalizability of the findings. Future research should enroll larger cohorts of myopes while ensuring uniformity of ocular and refractive characteristics to minimize confounding factors. Integrating multimodal imaging techniques, such as OCT and OCT angiography, will help correlate structural and functional changes in the retina. Longitudinal designs would further clarify how chronic lens use influences retinal electrophysiology over time.

## Conclusion

In conclusion, the tested defocus segment lens designs did not produce statistically detectable alterations in first order multifocal electroretinography parameters in structurally intact emmetropic eyes under brief exposure. Although the analytical framework demonstrated sufficient sensitivity to identify medium to large electrophysiological effects, the current design may lack resolution for subtle retinal adaptations. Future investigations incorporating larger cohorts, prolonged optical exposure, and nonlinear analytical techniques in ametropic populations will be necessary to determine whether clinically relevant myopia control mechanisms correspond to measurable electrophysiological modulation.

## Supplemental Information

10.7717/peerj.21766/supp-1 Supplemental Information 1 Statistically significant *post hoc* comparisons between variables *Post hoc* comparisons were performed using Tukey’s HSD test to assess differences in mean scores between variables. Only comparisons with statistically significant differences are summarized.

10.7717/peerj.21766/supp-2 Supplemental Information 2 STROBE checklist

10.7717/peerj.21766/supp-3 Supplemental Information 3 Raw dataset used for statistical analyses of variables across the study population The original raw dataset used for all analyses reported in the manuscript. No data filtering, transformation, or normalization has been applied. Variable definitions and measurement procedures are described in the methods section of the article.

10.7717/peerj.21766/supp-4 Supplemental Information 4 Raw dataset containing accommodation variables, used for statistical analysis of accommodative characteristics across the study population The raw dataset of ocular accommodation measurements used for all analyses presented in the manuscript. The data have not undergone filtering, transformation, or normalization. Definitions of each accommodative parameter and their corresponding measurement protocols are detailed in the ‘Methods’ section.

## Additional Information and Declarations

### Data Availability

The following information was supplied regarding data availability: The raw datasets are available in the Supplemental Files.
