# Supplementary material for: Multifocal electroretinography assessment of retinal responses to myopic defocus lenses
Source: PeerJ. 2026 Oct 5;14:e21766. doi: 10.7717/peerj.21766 (PMC13648215; doi:10.7717/peerj.21766)
Supplement: Supplementary file 1 [file peerj-14-21766-s001.docx]

Supplementary table 1: Summary of statistically significant mean score differences between variables identified through post hoc multiple comparisons using the Tukey HSD test.

| **Test Condition** | **Dependent Variable** | **Areas** | | **Mean Difference** | **Standard Error** | ***p-*value** | **95% Confidence Interval** | |
| --- | --- | --- | --- | --- | --- | --- | --- | --- |
|  |  |  |  |  |  |  | **Lower Bound** | **Upper Bound** |
| **No lens** | Positive latency (ms) | Ring 2 | Ring 5 | -1.6357 | 0.4700 | 0.008* | -2.954 | -0.317 |
|  |  | Ring 3 | Ring 5 | -1.5500 | 0.4700 | 0.013* | -2.869 | -0.231 |
|  | Voltage (nV/d^2^) | Ring 1 | Ring 2 | 18.6500 | 1.9018 | 0.000* | 13.314 | 23.986 |
|  |  | Ring 1 | Ring 3 | 28.5857 | 1.9018 | 0.000* | 23.250 | 33.922 |
|  |  | Ring 1 | Ring 4 | 32.4571 | 1.9018 | 0.000* | 27.121 | 37.793 |
|  |  | Ring 1 | Ring 5 | 34.1714 | 1.9018 | 0.000* | 28.835 | 39.507 |
|  |  | Ring 2 | Ring 3 | -18.6500 | 1.9018 | 0.000* | -23.986 | -13.314 |
|  |  | Ring 2 | Ring 4 | 9.9357 | 1.9018 | 0.000* | 4.600 | 15.272 |
|  |  | Ring 2 | Ring 5 | 13.8071 | 1.9018 | 0.000* | 8.471 | 19.143 |
|  |  | Ring 3 | Ring 5 | 5.5857 | 1.9018 | 0.036* | 0.250 | 10.922 |
| **Bifocal** | Positive latency (ms) | Ring 2 | Ring 5 | -1.3714 | 0.4707 | 0.038* | -2.692 | -0.051 |
|  |  | Ring 3 | Ring 5 | -1.4071 | 0.4707 | 0.031* | -2.728 | -0.086 |
|  | Voltage (nV/d^2^) | Ring 1 | Ring 2 | 18.5857 | 2.4961 | 0.000* | 11.582 | 25.589 |
|  |  | Ring 1 | Ring 3 | 28.6286 | 2.4961 | 0.000* | 21.625 | 35.632 |
|  |  | Ring 1 | Ring 4 | 32.4429 | 2.4961 | 0.000* | 25.439 | 39.447 |
|  |  | Ring 1 | Ring 5 | 34.2571 | 2.4961 | 0.000* | 27.253 | 41.261 |
|  |  | Ring 2 | Ring 3 | 10.0429 | 2.4961 | 0.001* | 3.039 | 17.047 |
|  |  | Ring 2 | Ring 4 | 13.8571 | 2.4961 | 0.000* | 6.853 | 20.861 |
|  |  | Ring 2 | Ring 5 | 15.6714 | 2.4961 | 0.000* | 8.668 | 22.675 |
| **Miyosmart**  **(0.00D)** | Negative latency (ms) | Ring 1 | Ring 3 | 1.0857 | 0.3196 | 0.010* | 0.189 | 1.983 |
|  | Positive latency (ms) | Ring 1 | Ring 5 | -1.4571 | 0.4310 | 0.010* | -2.666 | -0.248 |
|  |  | Ring 2 | Ring 4 | -1.2571 | 0.4310 | 0.038* | -2.466 | -0.048 |
|  |  | Ring 2 | Ring 5 | -1.7571 | 0.4310 | 0.001* | -2.966 | -0.548 |
|  |  | Ring 3 | Ring 5 | -1.5143 | 0.4310 | 0.007* | -2.724 | -0.305 |
|  | Voltage (nV/d^2^) | Ring 1 | Ring 2 | 17.4929 | 1.9058 | 0.000* | 12.146 | 22.840 |
|  |  | Ring 1 | Ring 3 | 26.7571 | 1.9058 | 0.000* | 21.410 | 32.104 |
|  |  | Ring 1 | Ring 4 | 30.5500 | 1.9058 | 0.000* | 25.203 | 35.897 |
|  |  | Ring 1 | Ring 5 | 32.1571 | 1.9058 | 0.000* | 26.810 | 37.504 |
|  |  | Ring 2 | Ring 3 | 9.2643 | 1.9058 | 0.000* | 3.917 | 14.612 |
|  |  | Ring 2 | Ring 4 | 13.0571 | 1.9058 | 0.000* | 7.710 | 18.404 |
|  |  | Ring 2 | Ring 5 | 14.6643 | 1.9058 | 0.000* | 9.317 | 20.012 |
|  |  | Ring 3 | Ring 5 | 5.4000 | 1.9058 | 0.047* | 0.053 | 10.747 |
| **Stellest**  **(0.00D)** | Negative latency (ms) | Ring 1 | Ring 2 | 1.0857 | 0.3480 | 0.022* | 0.109 | 2.062 |
|  |  | Ring 1 | Ring 3 | 1.3429 | 0.3480 | 0.002* | 0.367 | 2.319 |
|  | Positive latency (ms) | Ring 2 | Ring 5 | -1.7286 | 0.4752 | 0.005* | -3.062 | -0.395 |
|  |  | Ring 3 | Ring 5 | -1.4643 | 0.4752 | 0.024* | -2.798 | -0.131 |
|  | Voltage (nV/d^2^) | Ring 1 | Ring 2 | 19.2500 | 2.3308 | 0.000* | 12.710 | 25.790 |
|  |  | Ring 1 | Ring 3 | 29.2214 | 2.3308 | 0.000* | 22.682 | 35.761 |
|  |  | Ring 1 | Ring 4 | 33.0857 | 2.3308 | 0.000* | 26.546 | 39.625 |
|  |  | Ring 1 | Ring 5 | 34.8143 | 2.3308 | 0.000* | 28.275 | 41.354 |
|  |  | Ring 2 | Ring 3 | 9.9714 | 2.3308 | 0.001* | 3.432 | 16.511 |
|  |  | Ring 2 | Ring 4 | 13.8357 | 2.3308 | 0.000* | 7.296 | 20.375 |
|  |  | Ring 2 | Ring 5 | 15.5643 | 2.3308 | 0.000* | 9.025 | 22.104 |
| **Miyosmart**  **(-3.00D)** | Negative latency (ms) | Ring 1 | Ring 3 | 1.5214 | 0.3486 | 0.000* | 0.543 | 2.500 |
|  |  | Ring 1 | Ring 4 | 1.3929 | 0.3486 | 0.002* | 0.415 | 2.371 |
|  | Positive latency (ms) | Ring 2 | Ring 4 | -1.4429 | 0.4944 | 0.038* | -2.830 | -0.056 |
|  |  | Ring 2 | Ring 5 | -1.7214 | 0.4944 | 0.008* | -3.109 | -0.334 |
|  | Voltage (nV/d^2^) | Ring 1 | Ring 2 | 17.0857 | 2.1722 | 0.000* | 10.991 | 23.180 |
|  |  | Ring 1 | Ring 3 | 26.6714 | 2.1722 | 0.000* | 20.577 | 32.766 |
|  |  | Ring 1 | Ring 4 | 30.0071 | 2.1722 | 0.000* | 23.912 | 36.102 |
|  |  | Ring 1 | Ring 5 | 31.2714 | 2.1722 | 0.000* | 25.177 | 37.366 |
|  |  | Ring 2 | Ring 3 | 9.5857 | 2.1722 | 0.000* | 3.491 | 15.680 |
|  |  | Ring 2 | Ring 4 | 12.9214 | 2.1722 | 0.000* | 6.827 | 19.016 |
|  |  | Ring 2 | Ring 5 | 14.1857 | 2.1722 | 0.000* | 8.091 | 20.280 |
| **Stellest**  **(-3.00D)** | Negative latency (ms) | Ring 1 | Ring 3 | 1.5500 | 0.3164 | 0.000* | 0.662 | 2.438 |
|  |  | Ring 1 | Ring 4 | 1.0357 | 0.3164 | 0.014* | 0.148 | 1.924 |
|  |  | Ring 3 | Ring 5 | -1.1571 | 0.3164 | 0.005* | -2.045 | -0.269 |
|  | Positive latency (ms) | Ring 2 | Ring 5 | -1.5143 | 0.4179 | 0.005* | -2.687 | -0.342 |
|  |  | Ring 3 | Ring 5 | -1.2714 | 0.4179 | 0.027* | -2.444 | -0.099 |
|  | Voltage (nV/d^2^) | Ring 1 | Ring 2 | 16.1643 | 1.6986 | 0.000* | 11.398 | 20.930 |
|  |  | Ring 1 | Ring 3 | 25.0357 | 1.6986 | 0.000* | 20.270 | 29.802 |
|  |  | Ring 1 | Ring 4 | 28.4643 | 1.6986 | 0.000* | 23.698 | 33.230 |
|  |  | Ring 1 | Ring 5 | 29.8214 | 1.6986 | 0.000* | 25.056 | 34.587 |
|  |  | Ring 2 | Ring 3 | 8.8714 | 1.6986 | 0.000* | 4.106 | 13.637 |
|  |  | Ring 2 | Ring 4 | 12.3000 | 1.6986 | 0.000* | 7.534 | 17.066 |
|  |  | Ring 2 | Ring 5 | 13.6571 | 1.6986 | 0.000* | 8.891 | 18.423 |
|  |  | Ring 3 | Ring 5 | 4.7857 | 1.6986 | 0.049* | 0.020 | 9.552 |
