# Supplementary material for: Multifocal electroretinography assessment of retinal responses to myopic defocus lenses
Source: PeerJ. 2026 Oct 5;14:e21766. doi: 10.7717/peerj.21766 (PMC13648215; doi:10.7717/peerj.21766)
Supplement: Supplementary file 2 [file peerj-14-21766-s002.docx]

STROBE Statement—checklist of items that should be included in reports of observational studies

|  | Item No. | Recommendation | Page  No. | Relevant text from manuscript |
| --- | --- | --- | --- | --- |
| **Title and abstract** | 1 | (*a*) Indicate the study’s design with a commonly used term in the title or the abstract | 1-2 | "A prospective cross-sectional study enrolled 14 young, healthy emmetropic participants..." (Abstract, Materials & Methods section) |
|  |  | (*b*) Provide in the abstract an informative and balanced summary of what was done and what was found | 2 | "Aim: To examine the retinal electrical responses elicited by defocus segment lenses in emmetropic eyes using multifocal electroretinography (mfERG). Materials & Methods: A prospective cross-sectional study enrolled 14 young, healthy emmetropic participants aged 18 to 22 years... mfERG recordings were obtained using 6 different condition scenarios... Results: Retinal electrical activity varied significantly across hexagon ring regions (Rings 1–5) under all lens conditions. However, no significant differences were found when comparing the responses between the different lens types (p>0.05). Conclusion: The defocus segment lenses do not significantly alter retinal electrical responses in emmetropic eyes under short-term experimental exposure." (Abstract) |
| Introduction | | | |  |
| Background/rationale | 2 | Explain the scientific background and rationale for the investigation being reported | 2 | "Myopia has emerged as a significant global public health concern, with projections indicating that its prevalence will reach approximately 5 billion cases worldwide by 2050... Among these interventions, optical strategies have gained considerable attention... defocus segment lenses, such as defocus incorporated multiple segments (DIMS)... Clinical studies have demonstrated that DIMS lenses can reduce myopia progression by 52%... Despite these encouraging results, critical gaps remain in our understanding of how defocus segment lenses influence retinal function. Specifically, there is limited evidence on their impact on retinal electrophysiological responses..." (Introduction) |
| Objectives | 3 | State specific objectives, including any prespecified hypotheses | 3-4 | "The hypothesis that defocus segment lenses might elicit measurable short-term retinal electrophysiological changes is grounded in evidence demonstrating the rapid change in retinal response to optical defocus... we hypothesized that the specific optical defocus patterns generated by these modern lenses would produce a distinct, measurable signature on retinal function. Therefore, the present study was designed to evaluate these short-term retinal electrophysiological responses to defocus segment lenses using mfERG..." (Introduction); "The aim of this study is to examine the retinal electrical responses elicited by defocus segment lenses in emmetropic eyes using mfERG." (End of Introduction) |
| Methods | | | |  |
| Study design | 4 | Present key elements of study design early in the paper | 4 | "This cross-sectional study enrolled 14 visually healthy young adults (6 males, 8 females) aged 18 to 22 years (mean±SD: 20±1.46). Ethical approval was granted by the Institutional Review Board at King Saud University in accordance with the Declaration of Helsinki (approval number E-23-7783)." (Study design and subjects subsection) |
| Setting | 5 | Describe the setting, locations, and relevant dates, including periods of recruitment, exposure, follow-up, and data collection | 4-5 | "Ethical approval was granted by the Institutional Review Board at King Saud University in accordance with the Declaration of Helsinki (approval number E-23-7783)." (Affiliations on p.1 also indicate King Saud University, Riyadh, Saudi Arabia as primary institution); Note: Specific recruitment dates not reported in manuscript. |
| Participants | 6 | (*a*) *Cohort study*—Give the eligibility criteria, and the sources and methods of selection of participants. Describe methods of follow-up  *Case-control study*—Give the eligibility criteria, and the sources and methods of case ascertainment and control selection. Give the rationale for the choice of cases and controls  *Cross-sectional study*—Give the eligibility criteria, and the sources and methods of selection of participants | 4-5 | "Participants were included if they exhibited a spherical equivalent (SE) within the range of −0.50D to +0.50D and an uncorrected visual acuity (VA) of 6/6 or better. Exclusion criteria encompassed the presence of ocular or systemic disorders, a history of ocular or systemic trauma, current or past use of ocular or systemic medications, and impaired binocular vision. Additionally, participants with refractive parameters outside the specified SE range or with an uncorrected VA worse than 6/6 were excluded." (Study design and subjects subsection) |
|  |  | (*b*) *Cohort study*—For matched studies, give matching criteria and number of exposed and unexposed  *Case-control study*—For matched studies, give matching criteria and the number of controls per case | N/A | Not applicable: This was an unmatched cross-sectional study with repeated measures within participants. |
| Variables | 7 | Clearly define all outcomes, exposures, predictors, potential confounders, and effect modifiers. Give diagnostic criteria, if applicable | 5-6 | "Recordings were obtained six times for each participant, under six different experimental conditions: (1) no lens, (2) bifocal lens (plano +3.00D), (3) MiyoSmart Hoya lens (0.00D), (4) Essilor Stellest lens (0.00D), (5) MiyoSmart Hoya lens (−3.00D) and (6) Essilor Stellest lens (−3.00D)." (Data collection); "Retinal response data were stratified into five concentric hexagon rings based on angular distance from fixation: Ring 1 (2.5°), Ring 2 (7.5°), Ring 3 (14°), Ring 4 (22°), and Ring 5 (30°)... Comparisons between the six different conditions across these regions were based on the evaluation of negative latency (N1), positive latency (P1), and voltage." (Statistical analysis) |
| Data sources/ measurement | 8* | For each variable of interest, give sources of data and details of methods of assessment (measurement). Describe comparability of assessment methods if there is more than one group | 4-6 | "The mfERG recordings were obtained using the Diagnosys mfERG system (Diagnosys LLC, Lowell, MA) following the manufacturer's protocol. The mfERG stimulus array consisted of 61 non-scaled hexagons subtending 60 degrees horizontally and vertically, presented at a working distance of 33 cm... Each hexagon alternated between bright and dark states, following a pseudo-random binary m-sequence stimulation protocol... DTL electrodes (NEUROSPEC AG, Stan, Switzerland) with three adhesive electrodes were positioned at the center of the forehead and at the medial and lateral canthi. Recording DTL electrodes were placed along the lower eyelid margin following the instillation of proparacaine hydrochloride ophthalmic solution (0.5%) for corneal anesthesia. To ensure a stable cycloplegic effect throughout the recording period, two drops of 2.5% phenylephrine ophthalmic solution were administered to each eye at five-minute intervals." (Data collection subsection) |
| Bias | 9 | Describe any efforts to address potential sources of bias | 4-5 | "The sequence of conditions was randomized to minimize order effects... To ensure a stable cycloplegic effect throughout the recording period, two drops of 2.5% phenylephrine ophthalmic solution were administered to each eye at five-minute intervals. Residual accommodation was assessed using the push-up method 30 minutes after the initial drop administration. The experimental procedure was conducted in dim room illumination to minimize external light interference." (Data collection subsection) |
| Study size | 10 | Explain how the study size was arrived at | 8 | "Although the study included only 14 participants, the sample size was supported by a priori power analysis (G*Power; effect size d = 0.85, power = 0.70) as sufficient to detect physiologically relevant differences in mfERG responses. The repeated-measures design, in which each participant served as their own control across all six lens conditions, substantially reduced inter-subject variability and enhanced statistical sensitivity." (Discussion) |

Continued on next page

| Quantitative variables | 11 | Explain how quantitative variables were handled in the analyses. If applicable, describe which groupings were chosen and why | 5-6 | "Retinal response data were stratified into five concentric hexagon rings based on angular distance from fixation: Ring 1 (2.5°), Ring 2 (7.5°), Ring 3 (14°), Ring 4 (22°), and Ring 5 (30°) (Figure 1)." (Statistical analysis subsection) |
| --- | --- | --- | --- | --- |
| Statistical methods | 12 | (*a*) Describe all statistical methods, including those used to control for confounding | 5-6 | "Statistical analysis was performed using SPSS software version 29.0.1 (IBM SPSS Statistics Inc., Chicago, IL). Descriptive statistics, including means and standard deviations (SD), were computed for all quantitative variables. Comparative analyses were conducted using analysis of variance (ANOVA) or independent sample t-tests, depending on the number of independent variable categories. When necessary, the Tukey HSD correction for multiple comparisons was applied... Statistical significance was considered for p values < 0.05 in all analyses." (Statistical analysis subsection) |
|  |  | (*b*) Describe any methods used to examine subgroups and interactions | 5-6 | "To examine spatial differences in retinal responses, a one-way ANOVA was conducted to compare N1, P1, and voltage mean scores across five concentric hexagon rings... Post hoc comparisons were performed using Tukey HSD to assess mean score differences between variables..." (Comparison of electrical retinal activity between different retinal areas subsection) |
|  |  | (*c*) Explain how missing data were addressed | N/A | Not applicable: No missing data reported; all 14 participants completed all six experimental conditions. |
|  |  | (*d*) *Cohort study*—If applicable, explain how loss to follow-up was addressed  *Case-control study*—If applicable, explain how matching of cases and controls was addressed  *Cross-sectional study*—If applicable, describe analytical methods taking account of sampling strategy | 4 | **"Participants were recruited via purposive convenience sampling to ensure a homogeneous emmetropic cohort. No post-hoc weighting adjustments were applied, as the repeated-measures experimental design controlled for inter-subject variability”**  The relevant text from manuscript “Participants were included if they exhibited a spherical equivalent (SE) within the range of −0.50D to +0.50D and an uncorrected visual acuity (VA) of 6/6 or better. Exclusion criteria encompassed the presence of ocular or systemic disorders, a history of ocular or systemic trauma, current or past use of ocular or systemic medications, and impaired binocular vision. Additionally, participants with refractive parameters outside the specified SE range or with an uncorrected VA worse than 6/6 were excluded.” |
|  |  | (*e*) Describe any sensitivity analyses | N/A | Not performed or reported in the manuscript. |
| Results | | | | |
| Participants | 13* | (a) Report numbers of individuals at each stage of study—eg numbers potentially eligible, examined for eligibility, confirmed eligible, included in the study, completing follow-up, and analysed | 4,6 | "This cross-sectional study enrolled 14 visually healthy young adults (6 males, 8 females) aged 18 to 22 years..." (Methods); "An independent-samples t-test was conducted to evaluate potential differences in N1, P1, and voltage mean scores between the right and left eyes across the 6 experimental conditions. The analysis revealed no statistically significant differences between the two eyes under any condition (p > 0.05). Consequently, only data from the right eye were included in further analyses." (Results), All 14 participants completed all procedures; no attrition. |
|  |  | (b) Give reasons for non-participation at each stage | N/A | No exclusions after enrolment; all 14 participants completed all six experimental conditions. |
|  |  | (c) Consider use of a flow diagram | N/A | Flow diagram not included, though participant progression was straightforward with no dropouts. |
| Descriptive data | 14* | (a) Give characteristics of study participants (eg demographic, clinical, social) and information on exposures and potential confounders | 6 | "Age, SE, and axial length and accommodation characteristics did not significantly differ between male and female participants (p > 0.05) (Table 1)."; Table 1 provides mean±SD for age, SE, axial length, and accommodation amplitude stratified by sex. |
|  |  | (b) Indicate number of participants with missing data for each variable of interest | N/A | No missing data reported for any variable. |
|  |  | (c) *Cohort study*—Summarise follow-up time (eg, average and total amount) | N/A | Not applicable: Cross-sectional design with single-visit testing (no longitudinal follow-up). |
| Outcome data | 15* | *Cohort study*—Report numbers of outcome events or summary measures over time | N/A | Not applicable: Cross-sectional design. |
|  |  | *Case-control study—*Report numbers in each exposure category, or summary measures of exposure | N/A | Not applicable: Cross-sectional design. |
|  |  | *Cross-sectional study—*Report numbers of outcome events or summary measures | 6-7 | "One-way ANOVA was performed to compare mean N1, P1, and voltage values across the six experimental conditions. Results indicated no statistically significant differences in retinal electrical activity among the 6 conditions for any of the measured parameters (Figure 2)."; "Statistically significant differences in N1 mean scores were observed among the five retinal regions in all conditions except for the no lens (p = 0.06) bifocal lens condition (p = 0.208)... P1 mean scores differed significantly across the five retinal regions in all conditions (p < 0.05), and voltage mean scores also exhibited statistically significant differences across all conditions (p ≤ 0.01)." (Results section) |
| Main results | 16 | (*a*) Give unadjusted estimates and, if applicable, confounder-adjusted estimates and their precision (eg, 95% confidence interval). Make clear which confounders were adjusted for and why they were included | 6-7 | "Results indicated no statistically significant differences in retinal electrical activity among the 6 conditions for any of the measured parameters (Figure 2)."; All analyses reported unadjusted p-values from ANOVA/t-tests; No confounder adjustment performed as this was a controlled experimental study in homogeneous emmetropic participants. |
|  |  | (*b*) Report category boundaries when continuous variables were categorized | 5 | "Retinal response data were stratified into five concentric hexagon rings based on angular distance from fixation: Ring 1 (2.5°), Ring 2 (7.5°), Ring 3 (14°), Ring 4 (22°), and Ring 5 (30°)." (Statistical analysis subsection and Figure 1 caption) |
|  |  | (*c*) If relevant, consider translating estimates of relative risk into absolute risk for a meaningful time period | N/A | Not applicable: Study reports electrophysiological parameters (latencies, voltage), not risk estimates. |

Continued on next page

| Other analyses | 17 | Report other analyses done—eg analyses of subgroups and interactions, and sensitivity analyses | 7 | "Post hoc comparisons were performed using Tukey HSD to assess mean score differences between variables, and only the statistically significant results are summarized in supplementary table 1." (Results: Comparison of electrical retinal activity between different retinal areas) |
| --- | --- | --- | --- | --- |
| Discussion | | | | |
| Key results | 18 | Summarise key results with reference to study objectives | 7 | "Our findings reveal significant regional variability in retinal electrical activity across the posterior pole, consistent with prior studies, but no significant differences between lens types. These results contribute to understanding how optical interventions interact with retinal function in structurally intact eyes." (Discussion opening paragraph) |
| Limitations | 19 | Discuss limitations of the study, taking into account sources of potential bias or imprecision. Discuss both direction and magnitude of any potential bias | 8-9 | "However, the exclusion of myopes restricts the generalizability of the findings... It is also worth noting that recruitment of young adults meeting strict emmetropia criteria (SE −0.50D to +0.50D, uncorrected VA ≥6/6) was particularly challenging..." (Discussion); Limitations regarding sample size and external validity explicitly addressed. |
| Interpretation | 20 | Give a cautious overall interpretation of results considering objectives, limitations, multiplicity of analyses, results from similar studies, and other relevant evidence | 9 | "In conclusion, this study demonstrates that defocus segment lenses do not significantly alter retinal electrical responses in emmetropic eyes under short-term experimental exposure. The absence of measurable effects may reflect the retina's inherent resilience to defocus-induced perturbations in structurally intact eyes. However, these findings cannot be extrapolated to myopic populations, where structural alterations may amplify or modify lens-related electrophysiological responses." (Conclusion) |
| Generalisability | 21 | Discuss the generalisability (external validity) of the study results | 7-9 | "A fundamental methodological choice in this study was the recruitment of emmetropic participants to isolate the lens effects from confounding factors inherent to myopia... By contrast, emmetropes maintain a more uniform retinal architecture and lack significant preexisting pathology... However, the exclusion of myopes restricts the generalizability of the findings. Future research should enroll larger cohorts of myopes..." (Discussion) |
| Other information | |  | | |
| Funding | 22 | Give the source of funding and the role of the funders for the present study and, if applicable, for the original study on which the present article is based | N/A | The authors received no funding for this work. |

*Give information separately for cases and controls in case-control studies and, if applicable, for exposed and unexposed groups in cohort and cross-sectional studies.

**Note:** An Explanation and Elaboration article discusses each checklist item and gives methodological background and published examples of transparent reporting. The STROBE checklist is best used in conjunction with this article (freely available on the Web sites of PLoS Medicine at http://www.plosmedicine.org/, Annals of Internal Medicine at http://www.annals.org/, and Epidemiology at http://www.epidem.com/). Information on the STROBE Initiative is available at www.strobe-statement.org.
